# Supplementary material for: Differential locus coeruleus–hippocampus interactions during offline states
Source: eLife. 2026 Sep 4;14:RP109159. doi: 10.7554/eLife.109159 (PMC13544908; doi:10.7554/eLife.109159)
Supplement: Peer review history

# Editor report: eLife Assessment

**DOI:** 10.7554/eLife.109159.4.sa0

**Authors:** Adrien Peyrache

**Affiliations:** McGill University Canada

**Keywords:** Convincing, Important

This **important** study provides new insights into the neuronal dynamics of the locus coeruleus in relation to hippocampal sharp-wave ripples. Using high-temporal-resolution, multi-site electrophysiological recordings in rats, the authors present **convincing** evidence that ripples and locus coeruleus activity are inversely correlated to levels of arousal and noradrenaline tone is modulated by hippocampo-cortical coupling. Overall, the work will be of interest to neuroscientists studying large-scale brain coordination and memory processes.

# Referee report: Reviewer #1 (Public review)

**DOI:** 10.7554/eLife.109159.4.sa1

[Editor's note: This version has been assessed by the Reviewing Editor without further input from the original reviewers. The authors have addressed all concerns raised by the reviewers; no further changes are required at this point.]

Summary:

The manuscript by Yang et al. investigates the relationship between multi-unit activity in the locus coeruleus, putatively noradrenergic locus coeruleus, hippocampus (HP) sharp-wave ripples (SWR) and spindles using multi-site electrophysiology in freely behaving male rats. The study focuses on SWR during quiet wake and non-REM sleep, and their relation to cortical states (identified using EEG recordings in frontal areas) and LC units.

The manuscript highlights differential modulation of LC units as a function of HP-cortical communication during wake and sleep. They establish that ripples and LC units are inversely correlated to levels of arousal: wake, i.e. higher arousal correlates with higher LC unit activity and lower ripple rates. The authors show that LC neuron activity is strongly inhibited just before SWR detected during wake. During non-REM sleep, they distinguish "isolated" ripples from SWR coupled to spindles and show that inhibition of LC neuron activity is absent before spindle-coupled ripples but not before isolated ripples, suggesting a mechanism where noradrenaline (NA) tone is modulated by HP-cortical coupling. This result has interesting implications for the roles of noradrenaline in the modulation of sleep-dependent memory consolidation, as ripple-spindle coupling is a mechanism favoring consolidation. The authors further show that NA neuronal activity is downregulated before spindles.

Strengths:

In continuity with previous work from the laboratory, this work expands our understanding of the activity of neuromodulatory systems in relation to vigilance states and brain oscillations, an area of research that is timely and impactful. The manuscript presents strong results suggesting that NA tone varies differentially depending on coupling of HP SWR with cortical spindles. The authors place their findings back in the context of identified roles of HP ripples and coupling to cortical oscillations for memory formation in a very interesting discussion. The distinction of LC neuron activity between awake, ripple-spindle coupled events and isolated ripples is an exciting result and its relation to arousal and memory opens fascinating lines of research.

# Referee report: Reviewer #2 (Public review)

**DOI:** 10.7554/eLife.109159.4.sa2

Summary:

In this study, authors studied the synchrony between ripple events in Hippocampus, cortical spindles and Locus Coeruleus spiking. The results in this study together with the established literature on the relationship of hippocampal ripples with widespread thalamic and cortical waves, guided authors to propose a role for Locus Coeruleus spiking patterns in memory consolidation. The findings provided here, i.e. correlations between LC spiking activity and Hippocampal ripples, could provide basis for future studies probing the directional flow or the necessity of these correlations in the memory consolidation process. Hence, the paper provides enough scientific advance to highlight the elusive yet important role of Norepinephrine circuitry in the memory processes.

Strengths:

Authors were able to demonstrate correlations of Locus Coeruleus spikes with hippocampal ripples as well as with cortical spindles. Specific strength of the paper is in the demonstration that the spindles that activate with the ripples are comparatively different in their correlations with Locus Coeruleus than those which do not.

# Referee report: Reviewer #3 (Public review)

**DOI:** 10.7554/eLife.109159.4.sa3

This manuscript examines how locus coeruleus (LC) activity relates to hippocampal ripple events across behavioral states in freely moving rats. Using multi-site electrophysiological recordings, the authors report that LC activity is suppressed prior to ripple events, with the magnitude of suppression depending on ripple subtype. Suppression is stronger during wakefulness than during NREM sleep and least pronounced for ripples coupled to spindles.

The study is technically sound and addresses a timely and important question regarding how LC activity interacts with hippocampal and thalamocortical network events across vigilance states. While the findings are interesting, they remain observational in nature.

# Author comment: Author response

**DOI:** 10.7554/eLife.109159.4.sa4

**Authors:** Mingyu Yang, Oxana Eschenko

**Affiliations:** Max Planck Institute for Biological Cybernetics Tübingen Germany; Max Planck Institute for Biological Cybernetics Tübingen Germany

The following is the authors’ response to the previous reviews

**Public Reviews:**

**Reviewer #1 (Public review):**

Summary:

The manuscript by Yang et al. investigates the relationship between multi-unit activity in the locus coeruleus, putatively noradrenergic locus coeruleus, hippocampus (HP) sharp-wave ripples (SWR) and spindles using multi-site electrophysiology in freely behaving male rats. The study focuses on SWR during quiet wake and non-REM sleep, and their relation to cortical states (identified using EEG recordings in frontal areas) and LC units.

The manuscript highlights differential modulation of LC units as a function of HP-cortical communication during wake and sleep. They establish that ripples and LC units are inversely correlated to levels of arousal: wake, i.e. higher arousal correlates with higher LC unit activity and lower ripple rates. The authors show that LC neuron activity is strongly inhibited just before SWR detected during wake. During non-REM sleep, they distinguish "isolated" ripples from SWR coupled to spindles and show that inhibition of LC neuron activity is absent before spindle-coupled ripples but not before isolated ripples, suggesting a mechanism where noradrenaline (NA) tone is modulated by HP-cortical coupling. This result has interesting implications for the roles of noradrenaline in the modulation of sleep-dependent memory consolidation, as ripple-spindle coupling is a mechanism favoring consolidation. The authors further show that NA neuronal activity is downregulated before spindles.

Strengths:

In continuity with previous work from the laboratory, this work expands our understanding of the activity of neuromodulatory systems in relation to vigilance states and brain oscillations, an area of research that is timely and impactful. The manuscript presents strong results suggesting that NA tone varies differentially depending on coupling of HP SWR with cortical spindles. The authors place their findings back in the context of identified roles of HP ripples and coupling to cortical oscillations for memory formation in a very interesting discussion. The distinction of LC neuron activity between awake, ripple-spindle coupled events and isolated ripples is an exciting result and its relation to arousal and memory opens fascinating lines of research.

Weaknesses:

I regretted that the paper fell short of trying to push this line of idea a bit further, for example by contrasting in the same rats the LC unit-HP ripple coupling during exploration of a highly familiar context (as seemingly was the case in their study) versus a novel context, which would increase arousal and trigger memory-related mechanisms. Any kind of manipulation of arousal levels and investigation of the impact on awake vs non-REM sleep LC-HP ripple coordination would considerably strengthen the scope of the study.

Comments on revised version:

The authors have added methodological details to the results section after the first round of reviews, improving the manuscript readability. Some points might still be improved, for example, the authors use a delta/gamma ratio to track cortical states for example, but there is no methods section corresponding to this metric. Authors write that higher SI corresponds to a lower arousal state that is associated with "more synchronized cortical population activity, higher ripple rate and reduced LC neurons firing" but there are no references or analysis to support this statement, only examples showing changes in SI over a few minutes.

We thank Reviewer #1 for the positive evaluation of our study and for highlighting its strengths and potential avenues for future investigation.

We have specified in the Methods the calculation of SI as a delta/gamma ratio and provided the frequency ranges used for each band: “Artefact-free EEG signals were band-pass filtered using a Butterworth filter implemented in Matlab 2024a (MathWorks, Natick, MA). Subsequently, deltaband power (δ, 1–4 Hz), theta-band power (θ, 6–10 Hz), and the θ/δ power ratio were computed within contiguous 4-second epochs.”

We agree with the reviewer and have acknowledged in the Discussion that incorporating behavioral assays will be essential for achieving a mechanistic understanding of the observed network dynamics and their functional role in memory consolidation. Such experiments are beyond the scope of the present study but represent an important direction for future research. We have also revised the Discussion to avoid overstated claims and to ensure that our interpretation remains appropriately supported by the current data. Discussion (last paragraph): “Conducting behavioral assays before electrophysiological recordings, along with spatially and temporally precise modulation of LC activity during recording sessions, will be essential for achieving a mechanistic understanding of network dynamics and its functional role for memory consolidation in future investigations.”

**Reviewer #2 (Public review):**

Summary:

In this study, authors studied the synchrony between ripple events in Hippocampus, cortical spindles and Locus Coeruleus spiking. The results in this study together with the established literature on the relationship of hippocampal ripples with widespread thalamic and cortical waves, guided authors to propose a role for Locus Coeruleus spiking patterns in memory consolidation. The findings provided here, i.e. correlations between LC spiking activity and Hippocampal ripples, could provide basis for future studies probing the directional flow or the necessity of these correlations in the memory consolidation process. Hence, the paper provides enough scientific advance to highlight the elusive yet important role of Norepinephrine circuitry in the memory processes.

Strengths:

Authors were able to demonstrate correlations of Locus Coeruleus spikes with hippocampal ripples as well as with cortical spindles. Specific strength of the paper is in the demonstration that the spindles that activate with the ripples are comparatively different in their correlations with Locus Coeruleus than those which do not.

Weaknesses:

The claims regarding the roles of these specific interactions were mostly derived from the literature that these processes individually contribute to the memory process, without any evidence of these specific interactions being necessary for memory processes. There are also issues with the description of methods, validation of shuffling procedures and unclear presentation and the interpretation of the findings, which are described in points that follow. I believe addressing these weaknesses might improve and add to the strength of the findings.

Comments on revised version:

The authors addressed all of my major concerns during the revision. As a result, the study now provides convincing evidence as well as improved presentation of results, that makes this manuscript important to the broader field of neuroscience, beyond the specific sub-field.

We thank Reviewer #2 for the positive assessment of our work and for recognizing both its strengths and its potential to stimulate future research in this area. We agree that assessing memory function is essential for understanding how noradrenergic signalling influences the network mechanisms underlying memory consolidation. While such experiments are beyond the scope of the present study, we acknowledge this important limitation in the Discussion and identify it as a key direction for future research. Discussion (last paragraph): “Conducting behavioral assays before electrophysiological recordings, along with spatially and temporally precise modulation of LC activity during recording sessions, will be essential for achieving a mechanistic understanding of network dynamics and its functional role for memory consolidation in future investigations.”

We added more details in the Methods and expanded the Figure 4 legend to improve the results presentation.

**Reviewer #3 (Public review):**

This manuscript examines how locus coeruleus (LC) activity relates to hippocampal ripple events across behavioral states in freely moving rats. Using multi-site electrophysiological recordings, the authors report that LC activity is suppressed prior to ripple events, with the magnitude of suppression depending on ripple subtype. Suppression is stronger during wakefulness than during NREM sleep and least pronounced for ripples coupled to spindles.

The study is technically sound and addresses a timely and important question regarding how LC activity interacts with hippocampal and thalamocortical network events across vigilance states. While the findings are interesting, they remain observational in nature. Following revision, the manuscript has substantially improved in both presentation and interpretation of the results, and most concerns have been addressed satisfactorily. I therefore only have a few minor considerations that the authors may wish to explore further in the current study or in future work, as these directions could provide additional mechanistic insight and would likely be of considerable interest to the field.

The authors demonstrate clearly that tonic LC firing rates preceding ripples differ significantly between wake-associated ripples (highest LC firing), isolated ripples during NREM sleep (lower LC firing), and spindle-coupled ripples (lowest LC firing). They also appropriately note that baseline firing differences will naturally influence the magnitude of LC suppression, which they also observe (highest LC reduction for wake ripples, then isolated ripples and last spindle-coupled ripples).

However, this aspect could be explored further, as it may provide additional insight into the regulation of spindle-associated ripple events. Since LC activity appears to decline gradually prior to ripple occurrence (Suppl. Figure 2), it would be interesting to test whether this gradual reduction helps organize the emergence of isolated versus spindle-coupled ripples. For example, isolated ripples may occur during the initial phase of LC decline, whereas spindle-coupled ripples may preferentially emerge when LC activity reaches its lowest levels. Such a relationship could also be consistent with the stronger synchronization observed for spindle-ripple coupling.

Related to this point, it would also be informative to examine whether isolated spindles occur more randomly in time, whereas spindle-associated ripple events appear more temporally clustered. If a single isolated spindle occurs, the associated LC suppression might be more pronounced. In contrast, when multiple spindle-associated ripple events occur in succession, LC activity may already be reduced following the first event, resulting in smaller additional suppression preceding subsequent events. Exploring this possibility could help clarify how LC dynamics shape the temporal emergence of ripple-subtypes

We are grateful to Reviewer #3 for the positive evaluation of our manuscript and for the constructive comments highlighting the significance of our findings and their implications for future studies. We agree that a more comprehensive investigation of cross-regional coupling and its modulation by the LC–NE system represents an important and still insufficiently explored area of research. Further elucidating the complexity of these interactions will be essential for understanding how noradrenergic signalling shapes large-scale brain network dynamics across behavioral states. We acknowledge it in the Discussion: “A more comprehensive investigation of cross-regional coupling and its modulation by the LC–NE system represents an important and still insufficiently explored area of research. Further elucidating the complexity of these interactions will be essential for understanding how noradrenergic signaling shapes large-scale brain network dynamics across behavioral states.”

**Recommendations for the authors:**

**Reviewer #3 (Recommendations for the authors):**

Figure 4: It would be helpful to show the unshuffled data at the front (it is hidden partly behind the unshuffled data). Also, the unshuffled data are not introduced in the text for this figure. Would be helpful. Please also add color bars to improve interpretability.

To improve readability and facilitate interpretation, we revised Figure 4. Specifically, we (1) reordered the plots to present the unshuffled (ripple) data at the front; (2) expanded the figure legend to provide a more detailed description of the shuffling procedure; and (3) removed the unnecessary color fill from the box plots in panels B and C, while retaining the labels.

Figure 7: The color coding appears wrong in panel F (mean curves in F do not correspond to time traces in G). This should be checked and corrected if necessary.

We have corrected the colour coding in Figure 7.
